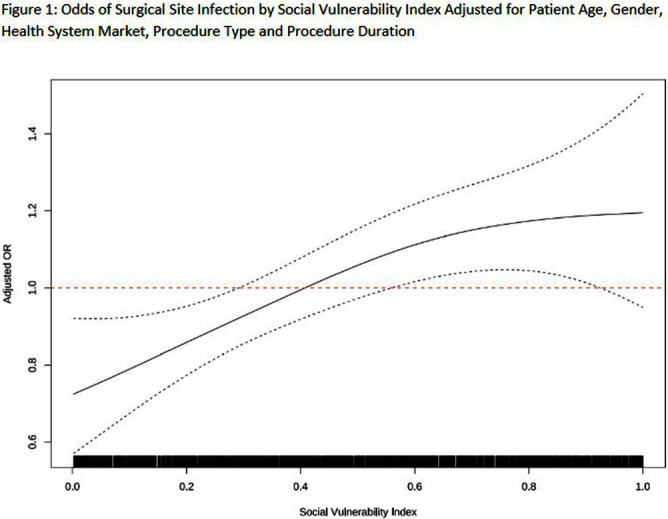# Exploring Socioeconomic Disparities in Surgical Site Infections

**DOI:** 10.1017/ash.2024.99

**Published:** 2024-09-16

**Authors:** Michael Dewitt, Caroline Reinke, Michael Inman, Werner Bischoff, Shelley Kester, Mindy Sampson, Anupama Neelakanta, Katie Passaretti

**Affiliations:** Wake Forest University School of Medicine; Atrium Health; Wake Forest School of Medicine; Stanford University; Carolinas HealthCare System

## Abstract

**Introduction:** Social disparities have been shown to impact a wide variety of healthcare outcomes. Surgical site infections (SSIs) are associated with substantial patient morbidity, but studies on the intersection of social disparity and SSI are limited. We sought to evaluate the association between SSI and the Center for Disease Control and Prevention’s social vulnerability index (SVI). **Methods:** Patients with National Health Safety Network (NHSN) procedure codes for colon, abdominal hysterectomy, hip prosthesis, knee prosthesis and spinal fusion surgeries were retrieved from the electronic medical records of 20 hospitals across 4 geographic markets. SSIs were identified by trained infection preventionists using NHSN definitions. Descriptive statistics were used for baseline demographic and clinical characteristics. Univariate logistic regression was performed to assess the association of demographic, clinical, and procedural factors with the outcome of SSI. Further univariate subgroup analysis was completed by procedure. To account for the nonlinear relationship between the social vulnerability index and SSIs, smoothing splines were used in a Bayesian hierarchical logistic regression model, with random effects to account for the different market practices. Nonlinear effects of procedure duration were also investigated while adjusting for the patient age, procedure type, and health system market. **Results:** 23,864 surgical procedures among 22,319 unique patients identified between 1 August 2022 and 31 August 2023. 96 patients with infection present at time of surgery were excluded. The study population was mostly white (74%) and female (65%) (Table 1). Less than 13% of the patients had more than one procedure during this time. In a univariate analysis, we found evidence of market and procedure effects, with colon surgery being associated with the highest odds of SSI. Procedure duration was significantly associated with SSI in both univariate and multivariable models, with a drastic increase in the odds of SSI for procedures > 150 mins. In the multivariable model we found that SVIs lower than 0.4 (95% CI 0.28 to 0.55) are associated with an adjusted odds ratio (aOR) < 1. (Figure 1) **Conclusions:** Our study shows that the relationship of social vulnerability and adverse outcomes is highly complex with nonlinear dynamics at play. After adjusting for procedure type, duration, patient age, gender and health system market the odds of SSIs increase sharply in patients with higher SVI until leveling off at an elevated risk.

**Figure:**
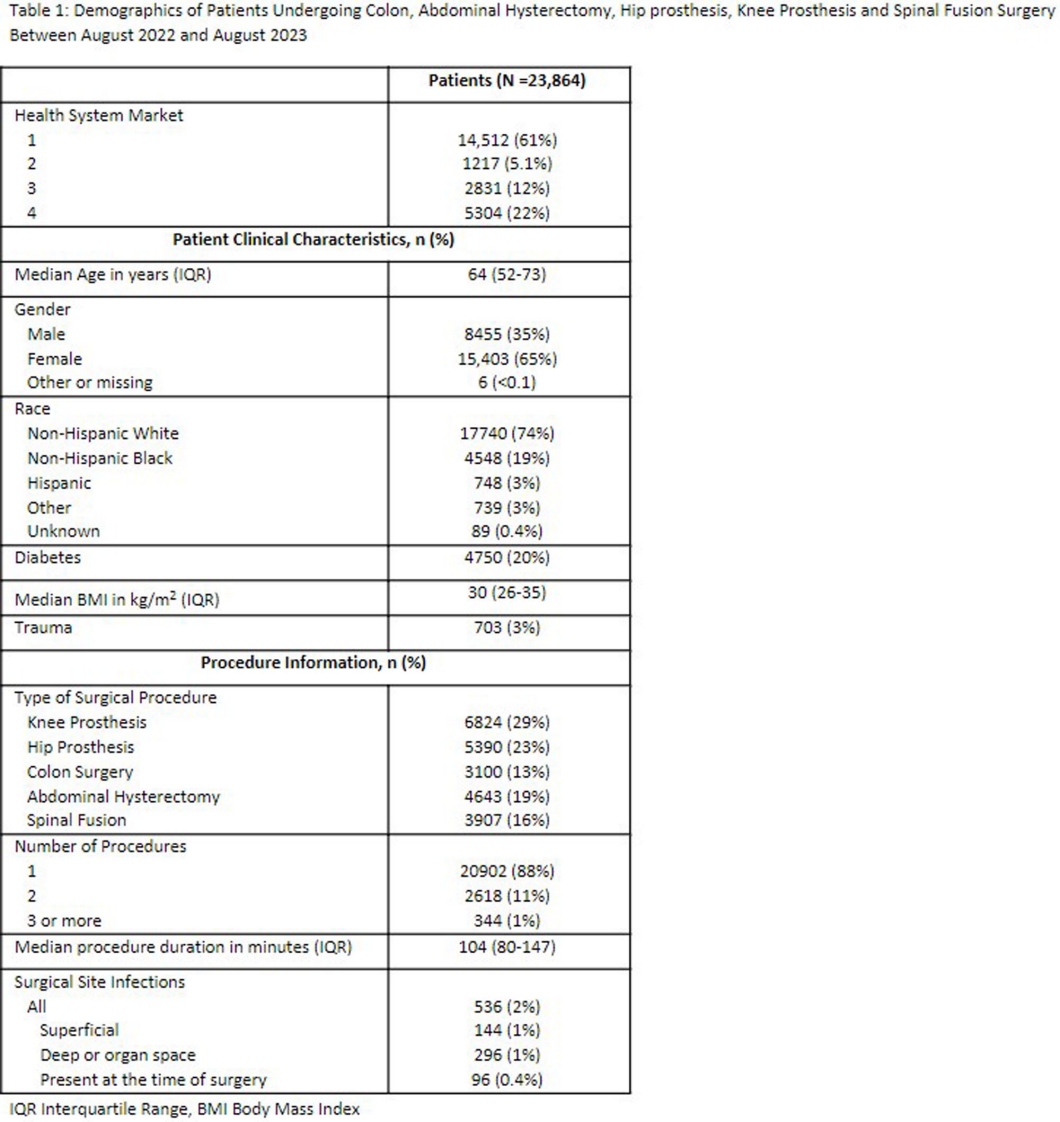

**Figure:**
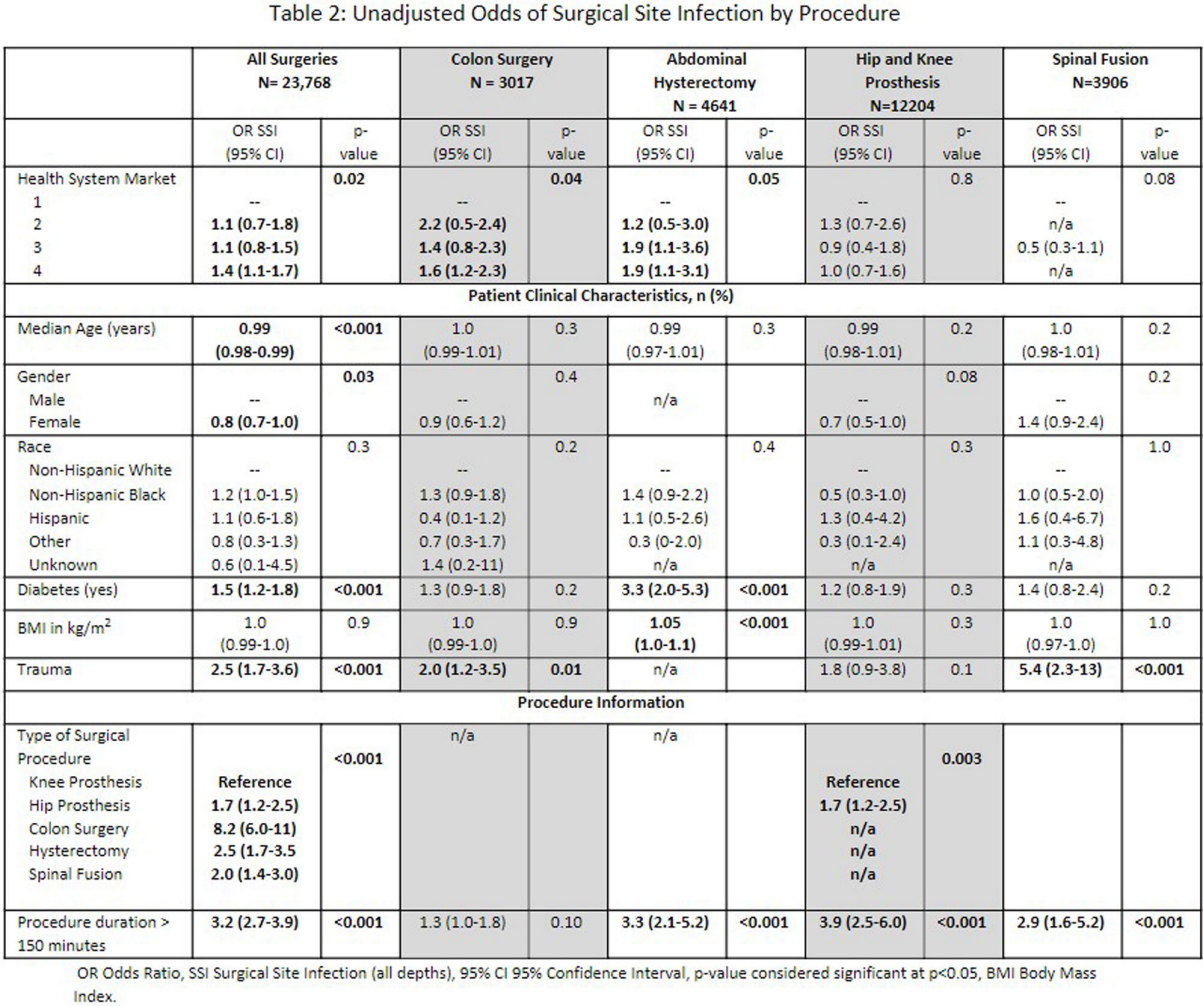

**Figure:**